# Case report: Fully endoscopic microvascular decompression for trigeminal neuralgia

**DOI:** 10.3389/fneur.2022.1090478

**Published:** 2023-01-11

**Authors:** Haotian Jiang, Dewei Zou, Pan Wang, Longwei Zeng, Jie Liu, Chao Tang, Gang Zhang, Xiaorong Tan, Nan Wu

**Affiliations:** Department of Neurosurgery, Chongqing General Hospital, Chongqing, China

**Keywords:** endoscopy, microvascular decompression, trigeminal neuralgia, suboccipito-retrosigmoid approach, functional neurosurgery

## Abstract

Microvascular decompression is safe, effective, and micro-invasive. Due to these advantages, it has become the mainstream treatment for trigeminal neuralgia, glossopharyngeal neuralgia, and hemifacial spasm. Initially, microvascular decompression was performed under a microscope, which limited the light source and visualization capabilities. With the development of endoscopic technology, the endoscope has been used in microvascular decompression, which further improved the visualization range and light source properties. The purpose of the present study was to investigate the efficacy of fully endoscopic microvascular decompression for the treatment of trigeminal neuralgia. In total, three patients with trigeminal neuralgia who underwent fully endoscopic microvascular decompression were evaluated. After surgery, the facial pain of all patients was significantly relieved. In addition, there were no obvious postoperative complications and no recurrence after 6 months of follow-up. These excellent surgical outcomes indicate that fully endoscopic microvascular decompression is an effective and safe method for the treatment of trigeminal neuralgia. Furthermore, it also shows that the endoscope presents advantages for use in microvascular decompression.

## Introduction

Trigeminal neuralgia (TN) is a common chronic neuropathic pain condition that presents with characteristic pain in one or more branches of the fifth cranial nerve (1). This condition is usually characterized by paroxysmal, transient, and intense pain and is described as a “shock” or “electric sensation” (2–5). Coughing, chewing, swallowing, gargling, or blowing may induce TN, which seriously affects the patient's quality of life. TN is more common in women than in men and its incidence rate increases with age (6). TN is divided into three types: classic, secondary, and special. Intracranial vascular compression of the trigeminal nerve root is the most common cause of classic TN (1). At present, drug therapy is the primary treatment for TN, and anticonvulsant carbamazepine is considered to be the first drug of choice (7, 8). Oxcarbazepine, lamotrigine, gabapentin, and pregabalin are also commonly used. However, drugs are not always effective for each patient and drug intolerance may occur. In these cases, surgical treatment may be considered. The mainstream surgical treatments include radiculotomy, radiofrequency thermocoagulation, glycerol rhizolysis, balloon compression, gamma-knife stereotactic radiosurgery, and microvascular decompression (MVD) (9–13). Among them, MVD is currently considered the most effective surgical treatment for classic TN. It has many advantages, as it is effective, minimally invasive, safe, and low-cost and has no recurrence (1, 2, 14, 15). Most MVD procedures are performed under a microscope, but not all offending vessels can be detected due to the microscope's limited field of vision. In contrast, the endoscope can provide brighter illumination and a panoramic view, which is more conducive to identifying the vessels of interest and evaluating the decompression effect (16–18). The present report describes three patients with TN who were successfully treated with fully endoscopic MVD. All patients had classical TN and a history of paroxysmal facial pain. The preoperative head MRI showed vascular compression of the trigeminal nerve. The case outcomes demonstrated that fully endoscopic MVD is an effective method for treating TN and that endoscopy presents significant advantages for use in MVD.

## Case report

### Case 1

**Figure 1:**
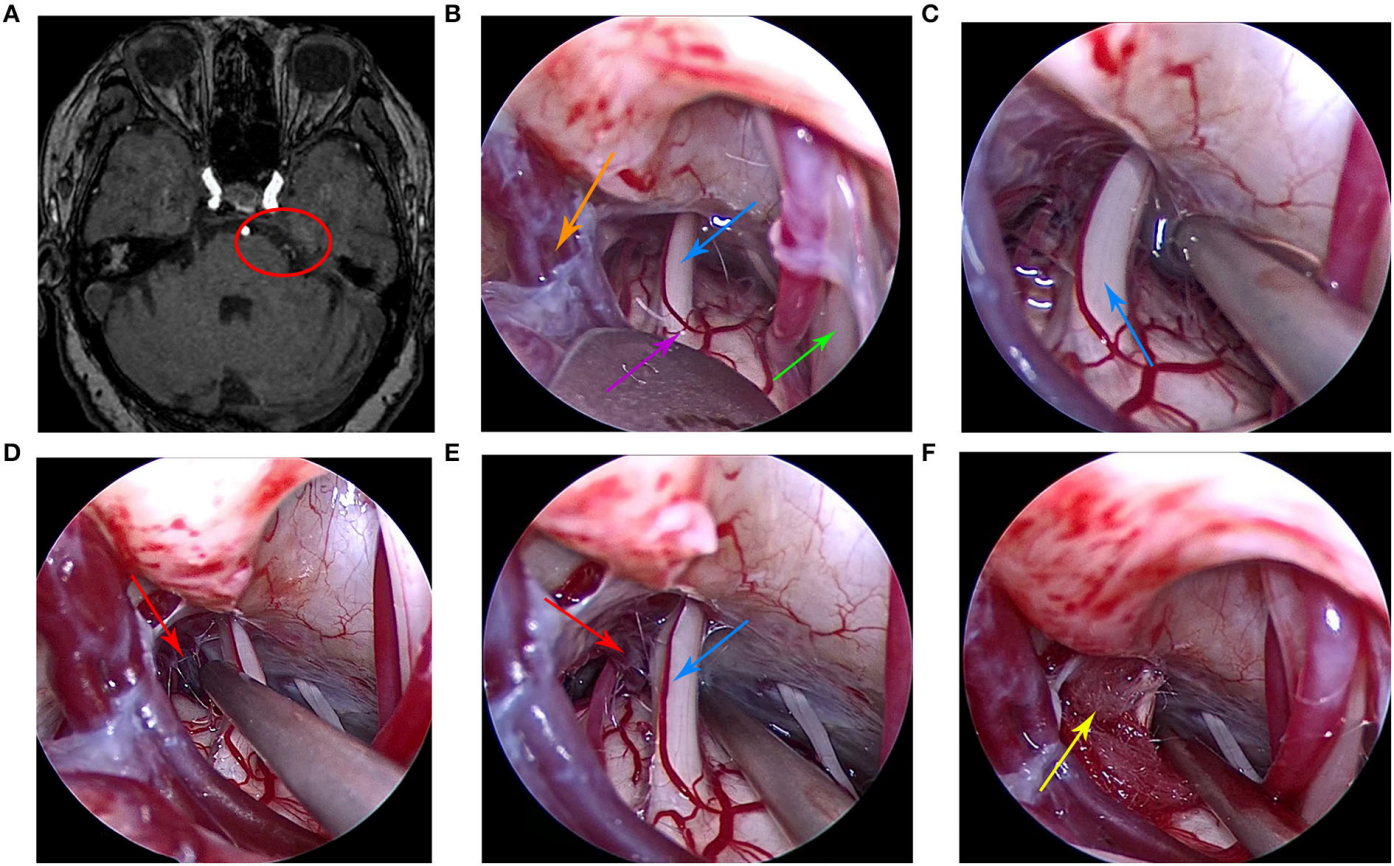
**(A)** Preoperative head magnetic resonance imaging of the patient. **(B)** Under the endoscope, the arachnoid was explored along the outer and upper margins of the cerebellum and subsequently cut to fully expose the trigeminal nerve. **(C)** At first, no obvious offending vessels were found. **(D)** After careful exploration, we found the offending small vein and arachnoid adhesions. **(E)** The petrosal vein branch and trigeminal nerve were clearly visible under the endoscopic view. **(F)** The Teflon pad was placed between the trigeminal nerve and the petrosal vein branch under the endoscopic view. The red circle indicates that the trigeminal nerve was compressed by the petrosal vein branch, the purple arrow indicates the brainstem, the orange arrow indicates petrosal vein, the red arrow indicates the petrosal vein branch, the blue arrow indicates the trigeminal nerve, the green arrow indicates the facial nerve, and the yellow arrow indicates the Teflon pad.

Case 1 was a 78-year-old woman who was admitted to the hospital in May 2020. The main clinical symptom was paroxysmal pain on the right side of the face over the course of 7 years, which was induced when brushing teeth and chewing. Each attack lasted for about 5 s. Hearing loss was present in the right ear. Neurological examination results were normal and pathological signs were negative. All physical examinations, including routine blood tests, assessments of liver and kidney, and evaluation of immune and blood coagulation functions, were within normal limits. The patient had no history of trauma or any family history of genetic disorders. She was treated at the departments of stomatology, otolaryngology, and neurology many times and was provided symptomatic treatment, such as analgesia and carbamazepine. However, the symptoms were not significantly relieved and continued to recur. The preoperative cranial MRI indicated that the right trigeminal nerve was compressed by small vessels (Figure 1A). Finally, the patient underwent fully endoscopic MVD *via* the suboccipito-retrosigmoid approach.

### Case 2

**Figure 2:**
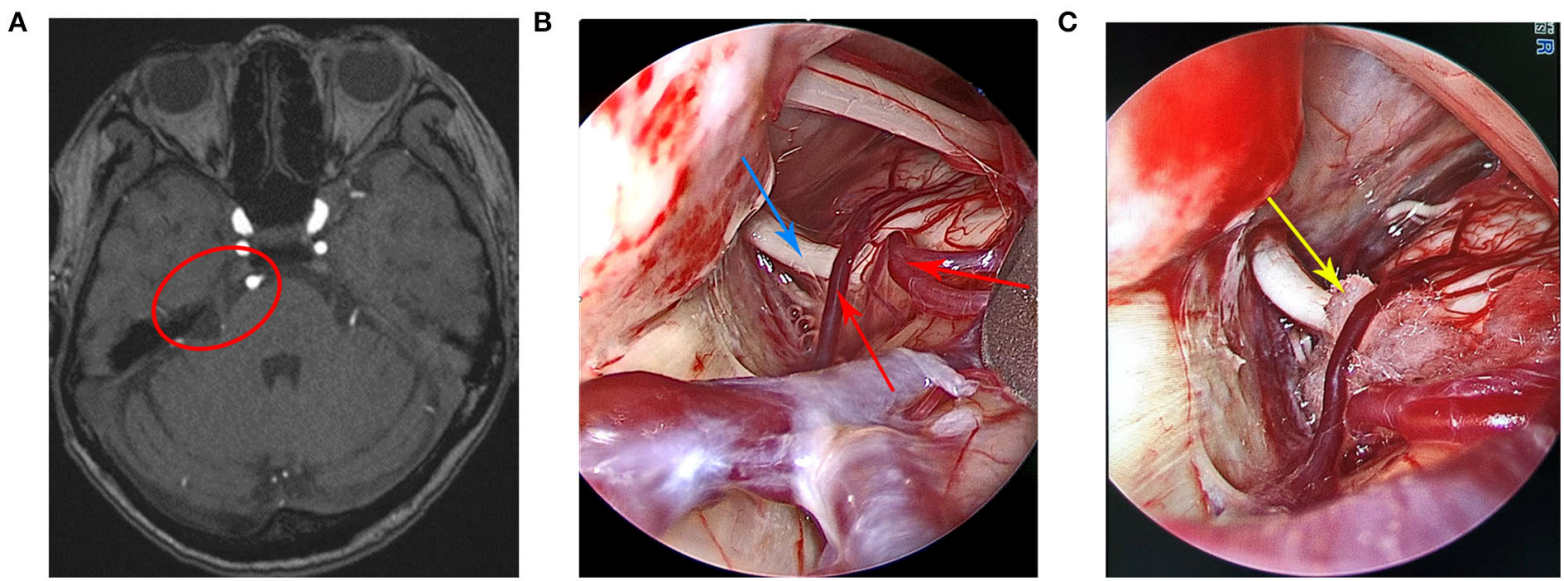
**(A)** Preoperative head magnetic resonance imaging of the patient. **(B)** A small artery, a vein that spanned the right trigeminal nerve, and the trigeminal nerve were clearly visible under the endoscopic view. **(C)** The Teflon pad was placed between the trigeminal nerve and the offending vessels under the endoscopic view. The red circle indicates trigeminal nerve compression by the offending vessels, the red arrow indicates the offending vessels, the blue arrow indicates the trigeminal nerve, and the yellow arrow indicates the Teflon pad.

Case 2 was a 60-year-old woman who was admitted to the hospital in November 2020. The main clinical symptom was paroxysmal discharge-like pain on the right side of the face over the course of 1 year, which was induced when brushing teeth and washing the face. Neurological examination was normal, and pathological signs were negative. The routine blood test outcomes, liver and kidney, and immune and blood coagulation functions were comprehensively examined, and the results showed that they were within the normal limits. The patient had no history of trauma or any family history of genetic disorders. The patient was diagnosed with right trigeminal neuralgia and was treated with carbamazepine and gabapentin without a positive effect. Preoperative cranial MRI showed that the right trigeminal nerve was compressed by blood vessels (Figure 2A). Finally, the patient underwent fully endoscopic MVD *via* the suboccipito-retrosigmoid approach.

### Case 3

**Figure 3:**
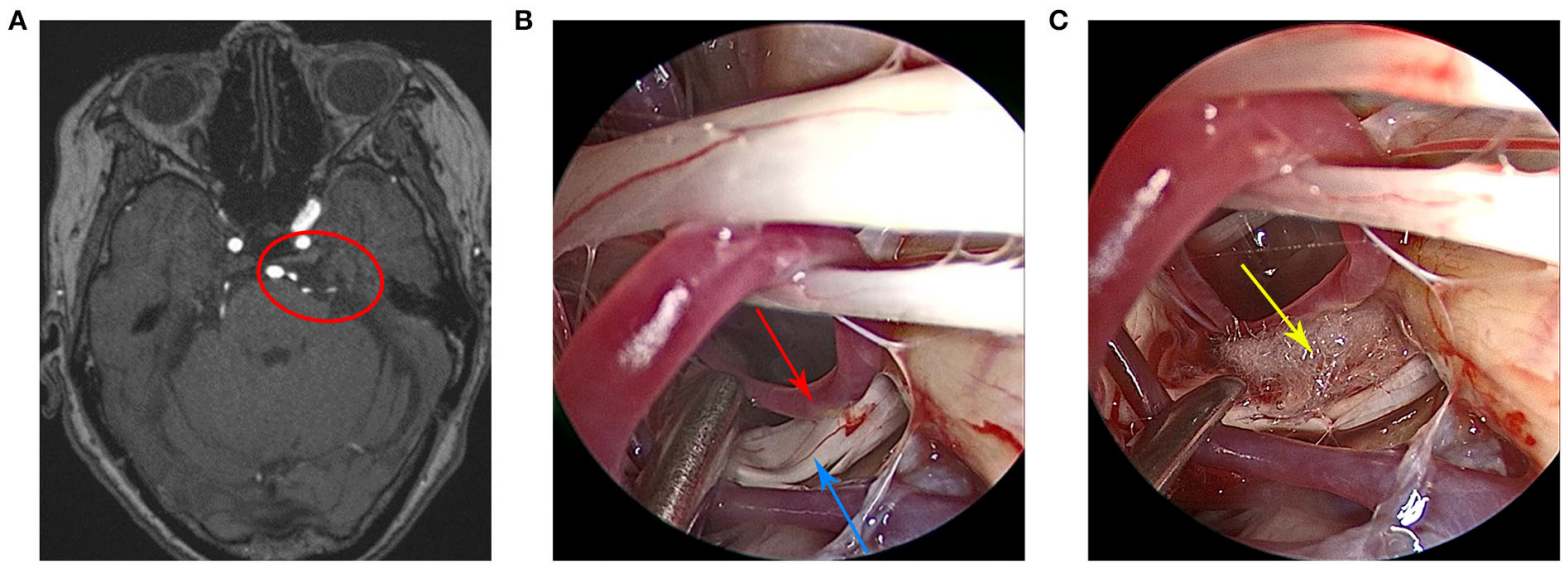
**(A)** Preoperative head magnetic resonance imaging of the patient. **(B)** The trigeminal nerve and the anterior cerebellar artery were clearly visible under the endoscopic view. **(C)** The Teflon pad was placed between the trigeminal nerve and the anterior cerebellar artery under the endoscopic view. The red circle indicates that the trigeminal nerve was compressed by the anterior cerebellar artery, the red arrow indicates the anterior cerebellar artery, the blue arrow indicates the trigeminal nerve, and the yellow arrow indicates the Teflon pad.

Case 3 patient was a 75-year-old woman who was admitted to the hospital in January 2021 due to the paroxysmal shock-like pain in the left side of the face over the course of 1 year. The pain was induced by drinking and eating. She received a comprehensive examination, which showed that the neurological examination results were normal and the pathological signs were negative. All laboratory tests, including routine blood tests, assessments of liver and kidney, and immune and blood coagulation functions, were within normal limits. The patient had no unambiguous history of trauma or any family history of genetic disorders. At first, the pain was relieved slightly after the oral carbamazepine treatment. However, the effect did not last and the pain became aggravated and recurred. In addition, the patient admitted to taking traditional Chinese medicine, which was also ineffective. Preoperative cranial MRI showed that the left trigeminal nerve was compressed by blood vessels (Figure 3A). Finally, the patient received fully endoscopic MVD *via* the suboccipito-retrosigmoid approach.

### Surgical procedure

All three patients underwent fully endoscopic MVD *via* the suboccipito-retrosigmoid approach. After general anesthesia, the patient was placed in a lateral decubitus position with the head drooping at 15° and rotated at 10° to the contralateral side. The neck was positioned slightly forward. The jaw was located about two transverse fingers away from the sternum, and the mastoid process on the surgical side was roughly parallel to the operating table at the highest position. First, a long transverse incision about 5 cm in length was made 1 cm below the star point behind the ear. The skin was cut, the muscle was dissected, and the bleeding was stopped. A bone window of about 3 cm × 3 cm was created using a grinding drill, and the transverse and sigmoid sinuses were fully exposed. Then, the dura mater was cut in an “X” shape, the arachnoid membrane was cut, and the cistern release was opened to release the cerebrospinal fluid to achieve full decompression. Under the endoscope, the arachnoid was explored along the outer and upper margins of the cerebellum and subsequently cut to fully expose the trigeminal nerve. Benefiting from the panoramic view provided by the endoscope, the compression of the trigeminal nerve by blood vessels was clearly observed. Then, the blood vessels in contact with the trigeminal nerve were separated and shifted, and a Teflon pad was placed between the offending blood vessels and the trigeminal nerve. In order to check for the complete separation of the offending vessels, decompression sufficiency, Teflon pad size and location suitability, and nerve and blood vessel damage, the endoscopy procedure was performed again. Finally, the dura mater and the edge of the bone window were suspended, the dura mater was repaired with a double layer of artificial dura mater to prevent cerebrospinal fluid leakage, the skull defect was repaired with four titanium plates and screws, and the muscle, subcutaneous tissue, and skin were sutured layer by layer.

## Results

### Overall results

Figures 1–3 show the intraoperative images of three patients, where the offending vessels can be clearly observed. The clinical symptoms of all patients were significantly relieved after the operation. The effectiveness rate was up to 100%. There were no subsequent medication requirements for the patients. Moreover, there were no obvious postoperative complications and no recurrence during the follow-up period in all cases. These surgical outcomes suggest that endoscopic MVD is a safe and effective method for the treatment of TN and that the endoscope has certain advantages and important application prospects for MVD.

### Individual case results

#### Case 1

Intraoperative endoscopy showed that the petrosal vein branch was adjacent to the right trigeminal nerve root. No arterial compression was observed around the trigeminal nerve. A Teflon pad was placed between the petrosal vein branch and the right trigeminal nerve. Postoperative facial pain symptoms were basically relieved, and hearing loss symptoms were slightly improved. There were no postoperative complications and no evidence of recurrence after 6 months of follow-up.

#### Case 2

During the operation, it was found that the root of the right trigeminal nerve was compressed by a small artery, and a vein spanned the right trigeminal nerve. Then, a Teflon pad was placed between the offending vessels and the right trigeminal nerve. After surgery, the patient's facial pain was completely relieved and there were no postoperative complications. There was no evidence of recurrence after 6 months of follow-up.

#### Case 3

Intraoperative endoscopy revealed that the left trigeminal nerve was compressed and deformed by the left anterior cerebellar artery. A Teflon pad was placed between the left anterior cerebellar artery and the left trigeminal nerve to separate them. As a result, the patient's facial pain was completely relieved without postoperative complications. There was no evidence of recurrence during the 6 months of follow-up.

## Discussion

Trigeminal neuralgia is a common chronic neuropathic pain condition that mainly manifests as paroxysmal, transient, and severe pain in the distribution area of the fifth cranial nerve, which seriously affects patient's quality of life. A description of TN was first recorded in the first century AD. Nicolas André first fully described the clinical symptoms of TN in 1756 and called it “tic douloureux” (19, 20). TN is also known as a “suicide disease” due to its severity and incidence rate (21). In 1773, John Fothergill first reported 14 patients with TN at the meeting of the London Medical Association (22). Since 1858, neurosurgeons have begun to explore a variety of surgical methods to treat TN. The early operation methods included neurectomy and neurolytic injection procedures (20). In 1901, Spiller and Frazier performed nerve root resection for patients with TN *via* the infratemporal approach. As a result, the pain was relieved but facial paralysis occurred (23). Later, Dandy cuts off the trigeminal nerve root using the cerebellar approach for TN treatment, which did not only achieve the good surgical results, but also significantly reduced the incidence of facial paralysis (24, 25). Dandy also suggested that most cases of TN were caused by compression of the trigeminal nerve by blood vessels, which is of great significance for the treatment of TN. In 1951, Taarnhøj was the first to perform a decompression operation on the trigeminal nerve root of a patient with TN instead of nerve root resection (26). In 1967, Jannetta was the first to come up with the most successful surgical method for TN-MVD and reported a case series of TN treated by MVD (27). Since then, MVD has been considered the most effective surgical method for the treatment of TN, especially classic TN, where the intracranial blood vessels compress the trigeminal nerve. Broggi et al. reviewed 250 patients with TN who underwent the MVD treatment. Approximately 75% of these patients experienced complete pain relief after surgery, and 15% relapsed during the long-term follow-up (28). Similarly, Mizobuchi et al. observed 166 patients with TN who received the MVD treatment. Their research results showed that after surgery, the complete pain relief rate was 79%, and the recurrence rate was about 20% over the course of a long-term follow-up (29). The above MVD procedure was performed under a microscope, and the surgical results showed that microscopic MVD was a relatively effective and safe treatment for these patients with TN. However, due to the light source and visualization capability limitations of the microscope, not all of the vessels of interest could be accurately located. To fully identify the offending blood vessels *via* a microscope, larger wounds and greater cerebellar contraction were required, which increases the possibility of postoperative complications. Due to the advantages of the wide field of vision, bright light source, the lack of obstruction in the field of vision, and operation flexibility, the endoscope has been widely used in various neurosurgery operations as well as MVD with good results (30, 31). Compared to the microscope, the biggest advantage of the endoscope is that it can accurately locate all of the vessels of interest. Chen et al. (17) found that about 14.74% of the offending vessels in 167 patients with TN were missed under a microscope and were only located when using an endoscope. Teo et al. (32) stated that the offending vessels of about 33% of patients with TN were difficult to see under a microscope, but easy to visualize under an endoscope. These findings suggest that an endoscope can increase the likelihood of identifying the offending vessels. Eby et al. (33) first treated TN with fully endoscopic MVD in 2002 and achieved good results. Bohman et al. reported that 47 patients with TN received a fully endoscopic MVD treatment. As a result, 94% of patients with TN experienced facial pain relief after surgery, and only one patient suffered from hearing loss (34). Sun et al. carried out fully endoscopic MVD in 20 patients with TN. All patients experienced pain relief after the operation. A total 16 patients had obvious pain relief, and four patients experienced good pain relief. None of the patients had complications (35). Meta-analysis by Zagzoog et al. compared the efficacy of MVD under both an endoscope and a microscope in the treatment of TN (16). The results showed that the remission and recurrence rates of the endoscopic and microscopic groups were similar. These values were 88 and 9%, as well as 81 and 14%, respectively. However, the endoscope group had significantly fewer postoperative complications than the microscope group, and the incidence rates were 8 and 19%, respectively. Lee et al. found that endoscopic MVD used for TN treatment had a lower incidence of complications compared to microscopic MVD, especially headaches. The incidence rates were 7 and 21%, respectively (36). Moreover, Lee et al. also found that endoscopic MVD was unlikely to cause complications after the TN treatment, especially headaches. The present study reported that three patients with TN were treated with fully endoscopic MVD. All of the offending blood vessels were clearly identified during the operation that was assisted by an endoscope, which significantly improved the surgery effect. After the operation, the patients' facial pain was significantly relieved without obvious postoperative complications. There was also no evidence of recurrence after 6 months of follow-up. These results indicate that fully endoscopic MVD was an effective and safe surgical method for TN in these patients. The following is a summary of the main reasons why endoscopic MVD can successfully treat TN. First, the endoscope provides good lighting, adequate visual angle, and wide field of vision during the operation, which does not only greatly improve the identification of blood vessels of interest and reduces the possibility of missing them, but also avoids damaging the surrounding brain tissues, blood vessels, and nerves and reduces postoperative complications. Second, after decompression, the endoscope can be used to evaluate whether the Teflon pad was correctly placed from multiple angles, whether the decompression was sufficient, and whether the surrounding tissues, nerves, and blood vessels were damaged. Finally, endoscopic MVD requires a relatively short surgical incision with less traction on brain tissue and cranial nerves and no significant cerebellar contraction, which means that the incidence of cerebellar contraction-related complications, such as cerebellar hemorrhage, infarction, swelling, and hearing loss, is relatively low (37). However, endoscopic surgery presents certain challenges for surgeons and requires a period of learning to be comfortable with this type of procedure. Sufficient training, experience, and up-to-date equipment can help to successfully master this technology. It should be noted that the present study had limitations due to a small number of patients included and a relatively short-term follow-up. In addition, it was designed as a non-randomized retrospective study and does not completely rule out potential selection bias.

## Conclusions

The present study described three TN cases that were successfully treated with fully endoscopic MVD. The surgical outcomes, including postoperative symptom relief, postoperative complications, and recurrence rate, indicate that fully endoscopic MVD may be a safe and effective method for TN treatment. Furthermore, the endoscope presents some advantages for use in MVD.

## Data availability statement

The original contributions presented in the study are included in the article/supplementary material, further inquiries can be directed to the corresponding author.

## Ethics statement

The studies involving human participants were reviewed and approved by the Committee Ethics of Chongqing General Hospital. The patients/participants provided their written informed consent to participate in this study. Written informed consent was obtained from the individual(s) for the publication of any potentially identifiable images or data included in this article.

## Author contributions

Study conception and design were contributed by HJ, DZ, PW, and LZ. Material preparation, data collection, and analysis were performed by HJ, JL, CT, GZ, XT, and NW. The first draft of the manuscript was written by HJ. All authors commented on the previous versions of the manuscript, read, and approved the final manuscript
